# Improving Risk Management by Learning from Adverse Events: Report on One Year of COVID-19 Vaccination Campaign in Verona (Northeastern Italy)

**DOI:** 10.3390/ijerph19063635

**Published:** 2022-03-18

**Authors:** Ilaria Tocco Tussardi, Stefano Tardivo

**Affiliations:** Department of Diagnostics and Public Health, University of Verona, 37134 Verona, Italy; ilaria.toccotussardi@univr.it

**Keywords:** clinical risk management, COVID-19 epidemiology, mass vaccination campaign, preparedness planning

## Abstract

Background: The COVID-19 mass vaccination campaign posed new challenges not only from a healthcare perspective, but also in terms of distribution, logistics, and organization. Managing clinical risk in off-site vaccination centers during a pandemic provided a new opportunity for the training and acquisition of competencies through continuous learning from adverse events. The aim of this report, based on a review of activity, was to identify the most recurrent and high-risk failures of the vaccination process in a mass vaccination center. Methods: Adverse events and near misses reported during the first 11 months of activity (February 2021–January 2022) in the mass vaccination center of Verona (Italy) were evaluated. Results: From 15 February 2021 to 17 January 2022 the center administered about 460,000 doses to the population and nine adverse events and one near miss were reported. Most of the events were errors in vaccine administration, either in principle, dosage, or timing with respect to the indicated schedule. All events had minor outcomes. Communication errors, inadequate training, and general organizational issues were the most recurrent factors contributing to the events. Conclusions: Risk mitigation during mass vaccination in temporary sites is an essential element of a successful vaccination campaign. The reporting of adverse events should be encouraged in order to obtain as much information as possible for a continuous improvement of the activity.

## 1. Introduction

Thirteen months after the first dose of COVID-19 vaccine was administered in Europe on 27 December 2020 (the so-called “V-Day”), 60.8% of the world’s population has received at least one dose of a COVID-19 vaccine, 9.98 billion doses have been administered globally, and 25.08 million are administered each day [1], with global numbers being updated on a daily basis by the World Health Organization (WHO) [2]. Since the start of the COVID-19 vaccines deployment in December 2020, the cumulative vaccine uptake in the total population in the EU/EEA has reached 70% for the complete primary course and 42.6% for an additional dose. However, progress differs across countries, with Bulgaria, Romania and Slovakia still reporting less than 50% of the total population having completed the primary vaccination course [3]. The COVID-19 vaccines validated for use by WHO and given Emergency Use Listing (EUL) are numerous. As of 12 January 2022, the vaccines that have obtained EUL are the Pfizer/BioNTech Comirnaty vaccine, the SII/COVISHIELD and AstraZeneca/AZD1222 vaccines, the Janssen/Ad26.COV 2.S vaccine, the Moderna COVID-19 vaccine (mRNA 1273), the Sinopharm COVID-19 vaccine, Sinovac-CoronaVac vaccine, the Bharat Biotech BBV152 COVAXIN vaccine, the Covovax (NVX-CoV2373) vaccine, and the Nuvaxovid (NVX-CoV2373) vaccine. All EU/EEA countries offer vaccination to those aged ≥12 years, and 23 countries are offering it to children 5−11 years of age [3]. Nonetheless, having licensed vaccines is just the first step in achieving global control of COVID-19. Vaccines need to be produced at scale, priced affordably, allocated rationally and made available where needed, and widely deployed in communities in order to maximize their impact and achieve widespread uptake [4]. The efforts made by national governments, international organizations, and private companies in developing and distributing vaccines were unprecedented. However, the first global mass vaccination campaign is encountering several obstacles, from inequalities in the distribution of vaccines in different parts of the world, to the need to re-evaluate schedules to provide consistently adequate protection, to the hesitation of individuals to receive the COVID-19 vaccination [5,6]. As the pandemic unfolds and variants emerge, the mass vaccination campaign is likely to go through new phases, adapting and modifying to contingencies, and finding new solutions to unresolved and emerging issues. From the perspective of optimizing resources to achieve the best results, deployment and allocation are among the crucial actions of the vaccination challenge, along with the development and production steps [4]. A robust coordination of data, infrastructure, communications and transport was required everywhere to enable an efficient distribution and administration of doses, both regionally and locally. In Italy, this was achieved through the support of multilateral initiatives to ensure timely global access. One of the major actions taken to maximize the availability of vaccines where needed was the incorporation of public and private halls (e.g., fairs, sports stadiums, malls) for temporary use as mass vaccination sites as part of a significant public-private engagement. This operation required an intense and innovative effort of logistical adaptation and rationalization of spaces in order to create rapid, effective, and safe vaccination pathways. In these new settings, managing clinical risk was made challenging by the unprecedented scenario, the rapid evolution of events, as well as the high media attention being paid to the mass vaccination campaign. The aim of this descriptive report was to identify the most recurrent and high-risk failures of the COVID-19 vaccination process in the mass vaccination center of Verona (Italy) by reviewing the adverse events that were reported during the first 11 months of activity. The analysis helped to identify some of the organizational issues that proved most impactful in the vaccination activity, as well as to highlight the most pressing risks associated with such a critical process.

## 2. Materials and Methods

### 2.1. Settings

The COVID-19 mass vaccination center (MCV) for the population of Verona (northeastern Italy, around 921,500 inhabitants) was established on 15 February 2021 in one of the pavilions of the city’s exhibition venue. The hall was divided into functional sectors (reception prior to entering the MCV, check in with temperature control, pre-vaccination medical evaluation, vaccination, post-vaccination observation, data registration and appointments) in order to create a rational user route. From 1 July 2021, vaccination activities formally fell under the management of the University Hospital of Verona (‘Azienda Ospedaliera Universitaria Integrata’, AOUI). Until 30 August 2021, the working sessions (two each day covering 12 h of activity, seven days a week) were organized with only one type of vaccine available to the population for the day, unless specific indications were given after a pre-vaccination medical evaluation. This was decided on in order to reduce the risk of administration errors. As the vaccination campaign continued, free access to the MCV was first opened to certain categories of people (e.g., the elderly) and then to the general population. The planning of the activities involved physicians, nurses, administrative staff and engineers from the local social-health unit and the University Hospital of Verona, alongside staff from the Municipality of Verona, the Italian Army, 500 municipal police officers and 5900 volunteers. As of 8 September 2021, the vaccination service was transferred to the University Hospital of Verona, in a venue that was specifically converted to host the mass vaccination campaign. With the increase in the number of requests for vaccinations, which was also due to the introduction of more stringent regulations in terms of vaccination coverage, in December 2021 two additional vaccination spaces were set up in the hospital, one specifically aimed at the pediatric population (<12 years of age). Overall, as of 15 January 2022, around 3000 vaccines are administered daily by the University Hospital.

### 2.2. Methods

We evaluated the reports of adverse events and near misses that occurred during the vaccination activity in the above-described settings and that were received by the Risk Management Unit of the University Hospital of Verona. Adverse events (AE) are defined as ‘unexpected events related to the care process and resulting in unintentional and undesirable harm to the patient. On the other hand, a near miss (NM) is ’an error that has the potential to cause an adverse event (a harm), which does not occur by chance or because it is intercepted or because it has no adverse consequences for the patient’ [7]. The reports were made using a special form in use at AOUI for incident reporting and analysis of AE and NM, in accordance with regulations of the Veneto Region [8]. The form is designed to collect the following information: place, date and time of event; qualification of reporting staff; type of event (with possibility of brief description); level of severity (0–5); factors contributing to the event (factors related to patient, communication, staff, organization, environment, IT, medical devices, drugs); factors that reduced severity; actions for improvement; further investigation/evaluation of the patient; the possibility of the recurrence of similar events; and the measurement of the detectability of error. The evaluation period was 11 months (15 February 2021–17 January 2022).

## 3. Results

During the eleven months considered, almost 460,000 vaccine doses were administered by the MCV to the population. Specifically, around 336,000 doses were administered from 15 February to 8 September 2021, and 113,000 additional doses until 17 January 2022. In this time period, nine reports were received by the MCV describing nine AEs (one reporting two different events) and one NM. The reports were transmitted on an average of three days after occurrence, except for one which was detected when a second AE occurred to the same patient (60 days after). All nine of the AEs were classified as level of severity 1 (the lowest). Detailed information including a brief description of the event, factors contributing, and improvement actions taken, are displayed in Table 1. None of the AEs required particular immediate action, except for an additional medical assessment in some cases. Only in one case did early detection reduce the severity of the event (AE 11 November 2021). One event was followed by a claim for compensation from the citizen involved. For all the events, recurrence was estimated as low or very low, and detectability of failure as high or very high. Additional information on severity levels of AEs, recurrence of events, and detectability of errors are reported in Table 2.

## 4. Discussion

The implementation of a mass vaccination campaign is a complex decision-making process, and is dependent on the country specific context. The demand to start the vaccinating the population as quickly and extensively as possible, and to progressively increase the vaccination capacities, found its answer in Italy in a new collaboration between local and state health authorities and private property owners to support the creation of temporary vaccination sites by changing the use of buildings. Guidelines were already available for organizations to implement vaccination campaigns in off-site locations, especially for seasonal influenza campaigns, but they referred mainly to US contexts [9,10,11]. The logistical obstacles and potential risks associated with the use of venues in an alternative manner were important and required an ad hoc risk assessment. The success of these efforts relied heavily on the collaboration and coordination between local and state health authorities, healthcare organizations, logistics experts, and healthcare and volunteer workforces. Risk mitigation planning for the temporary use of venues as COVID-19 vaccination sites required property, premises, COVID-19 safety, clinical, and other considerations. Characteristics considered and reviewed for each temporary site included size, location (urban, suburban, rural), duration of use, distribution and availability of space, road access and parking, accessibility for the disabled, and of course the expected number of vaccines to be delivered, all in light of foreseeable clinical risks. The Centers for Disease Control and Prevention (CDC) has provided guidelines for implementing vaccination clinics that are held at temporary or off-site locations during the COVID-19 pandemic, updating existing provisions in light of specific needs, including physical distancing, personal protective equipment, and enhanced sanitation efforts [12]. These guidelines have also been followed in our center, in addition to the guiding principles for holding safe vaccination clinics, and to the guidelines developed by vaccine manufacturers.

Given the number of doses administered in the period considered, we observed a very low incidence of adverse events, an average of one per month, with levels of severity that were never alarming, suggesting a fairly good control of risks and an activity that could be defined overall as safe, despite the initial accelerated implementation and also the subsequent increase recorded in response to the government’s dispositions [13]. Nonetheless, it must be noted that the distribution of reports over time was not homogeneous, being mainly concentrated in April, May and June 2021. The uneven distribution of reports may be attributable to various elements. We can hypothesize that during the first two months of activity the level of awareness of the importance of reporting was not optimal. This was emphasized progressively and especially after the management of the centre changed over to AOUI. The attention paid by the activity coordinators to the reporting activity also increased over time.

With regard to the double reporting on 18 June, after a specific review of the activity on that day we identified the high flow of people and the high number of newly-introduced personnel in the working groups as specific factors contributing to the events.

The adverse events detected had an impact on the functioning of the MCV through the implementation of improvement actions, e.g., changes in user flows, forms, and vaccine labelling. It is also important to remember that from September onwards there was a change in the setting, which greatly modified the characteristics of the activity from a logistical and organizational point of view; although the flows were still exceptionally high, the setting was more similar to an outpatient clinic.

From the evaluation of the events reported, it emerges that the largest proportion of adverse events are errors in vaccine administration, whether in principle, dosage or timing with respect to the indicated schedule. Fortunately, at the moment these events do not have any clinical consequences for the individuals involved, but they do constitute episodes with a very high potential for conflict between users and operators, as well as having media implications. The factors that may contribute to such events are various, as reported in Table 1, but most often they involve errors in communication and reading of documentation, combined with insufficient training of operators and issues related to the general organization. Our finding is supported by results from a priori analyses of the various stages of the mass vaccination process which identified pre-vaccination screening as one of the processes with the highest risk of failure [14]. In this area, the failures with the highest risk priority number were ‘Failure to investigate clinical history’, ‘Erroneous medical details provided by user’ and ‘Failure to assign correct type of vaccination based on contraindications derived from medical history’. The other process at greater risk of failure indicated by the study was the post-vaccination observation, for which in our experience we did not detect major criticalities with regard to some of the potential risks highlighted (e.g., ‘Inadequate management of adverse reactions during observation period’), while for others (e.g., ‘Users leave mass vaccination centre before completing the required observation period’) we can agree this is an event that if regularly reported would probably have been one of the most frequent adverse events. A further study of organisational and process risks highlights as critical points the regional supply logistics chain, vaccination sites, appointment management and the vaccine-COVID information system [15]. The stage relating to vaccination sites and appointment management was the one where the majority of the identified risks were. The analysis also emphasised the problem of overwork as the most common risk to which health workers involved in vaccination activities are subjected, which can cause disaffection and thus entail greater risks for the entire organisation. This aspect, although already explored in other contexts, will most likely be addressed by specific investigations in the future.

At present, there are no similar reports in the literature that would allow comparisons to be made with other temporary COVID vaccination sites in the country. Reports published by the Institute for Safe Medication Practices (ISMP) are available which contain US data on COVID-19 vaccine errors voluntarily reported to ISMP [16,17]. In the report of the first four months of vaccine activity, of the errors reported to ISMP, 20% represented an incorrect dose being given (lower or higher than authorized), 11% constituted a mixed vaccine series, and another 11% involved the wrong volume of the diluent being administered; 9% were classified as the vaccine being given at the incorrect time interval; and 17% were related to the wrong age group, but with indications that were later revised (less than 16 for Pfizer-BioNTech vaccine; less than 18 for Moderna/Johnson&Johnson vaccines) [17]. As an indicator of the emerging importance of this type of adverse event in vaccine activities, authorities have made available several recommendation documents on vaccine administration errors which emphasize the importance of informing the recipient of the vaccine administration error, reporting the administration error, even those not associated with adverse events, and determining how the error occurred and implementing strategies to prevent it from happening again. Recently, the CDC issued specific interim guidance for revaccination when an error occurs with a COVID-19 vaccine [18]. The guidance provides direction for various types of administration-related events, from site and age group errors to errors in formulation and dosing, storage and dilution, and administration intervals. For each scenario, the guidance provides an indication of whether or not to repeat the dose, either immediately or after a given interval, and to use the age-appropriate COVID-19 vaccine and formulation after the event. From these elements, we can reasonably draw the conclusion that administration error is likely to be the most frequent adverse event occurring in temporary vaccination centers, which is also in agreement with the findings of this report.

### Limitations

This short paper does not addresses adverse reactions to vaccine administration, for which there is a specific monitoring system in Italy [19], but only adverse events detecting potential organizational criticalities. The number of adverse events and near misses reported may not represent the actual number of events that occurred in the center, and this might be attributable due an under-reporting that cannot always be effectively controlled, and also due to the demanding nature of vaccine activity. Furthermore, with regard to administration errors, no national data are available.

## 5. Conclusions

The COVID-19 mass vaccination campaign is a key component for restoring the functionality of societies, reducing COVID-related diseases and fatalities, and progressively normalizing health system activities. Mass vaccination sites need to implement strategies to mitigate risks as much as possible in order to achieve the highest level of success. Albeit reactive, monitoring of failures remains an essential tool for the identification of risks and the improvement of the quality of activities. As mass vaccination activities continue, more data will be available to help identify the most prominent criticalities, allowing measures to be taken proactively to prevent their occurrence.

## Figures and Tables

**Table 1:** Adverse events and near misses reported by the MVC of Verona from 15 February 2021 to 17 January 2022.

Date	Description	Factor(s) Contributing	Improvement Action(s)
Adverse event			
7 April 2021, 5 PM	A patient is administered VaxZevria but is mistakenly informed she has been given Comirnaty vaccine.	Inadequate communication. Inexact reading of documents. High flow of users.	Organization change Education and training.
30 April 2021, 7 PM	A patient is mistakenly administered VaxZevria instead of Comirnaty vaccine. Failure of user to comply with route instruction and of screening at vaccination box.	Inadequate communication. Logistics and organization aspects contributing to risk.	Internal audit. Organization change. Education and training.
31 May 2021, 9 AM (occurrence 30 April 2021, 10 AM)	Booking of second dose following the schedule of VaxZevria for a patient vaccinated with a first dose of Comirnaty vaccine.	Reduced patient autonomy. Inadequate communication. Difficulties in following procedures.	Internal audit. Education and training.
31 May 2021, 10 AM	A patient is mistakenly administered Moderna instead of Comirnaty vaccine. Failure of screening at entrance (Moderna day) and of pre-vaccination screening.	Inexact reading of documents. Newly-introduced personnel. Logistics and organization aspects contributing to risk.	Internal audit. Education and training.
18 June 2021, 2 PM	Wrong number of vaccine batch communicated to the operators. Error detected at the end of the session.	Inexpert reading of documents. New group and newly-introduced professionals.	Education and training.
12 July 2021, 10 AM	A patient is administered a second dose of Comirnaty (first dose VaxZevria) under medical prescription without an indication for heterologous vaccination.	Inadequate analysis of clinical documents. Newly-introduced professionals.	Organization change.
28 September 2021, 10 AM	A patient is admitted to the MCV (free access) to receive the second dose of Comirnaty (Moderna day). The dose is labelled differently and left on the cart next to the Moderna vaccines. The physician mistakenly takes and administers a dose of Moderna.	Failure to read label. Lack of supervision. High turnover of staff.	Organization change.
11 November 2021, 4 PM	Thirteen doses of Comirnaty vaccine are found unattended in a cart at the end of a working session.	Newly-introduced staff. Logistics and organization aspects contributing to risk.	Internal audit. Organization change.
29 December 2021, 4 PM	A pediatric user booked at the MCV is mistakenly administered an adult dose of Comirnaty.	Inadequate communication between users and staff and between professionals.	Internal audit. Organization change.
Near miss			
18 June 2021, 9 AM	A patient booked for VaxZevria is admitted to the MCV on a Comirnaty day. Failure of screening at entrance and at pre-vaccination site. Error is detected at the vaccination box.	Inadequate communication between professionals. High flow of users. Inexpert reading of documents. New group and newly-introduced professionals.	Education and training.

**Table 2:** Additional definitions related to the classification of adverse events and near misses.

Level	Description
0	Event not occurred, near miss
1	Minor outcome (extra observations or monitoring/further examination by doctor/no harm occurred or minor harm not requiring treatment)
2	Moderate outcome (extra observations or monitoring/additional medical examination/minor diagnostics/minor treatment)
3	Moderate to significant outcome (extra observations or monitoring/additional medical examination/diagnostic investigations/need for treatment with other medications/surgery/cancellation or postponement of treatment/transfer to other operative unit not requiring prolongation of hospital stay)
4	Significant outcome (admission to hospital or prolongation of hospital stay/conditions remaining at discharge)
5	Severe outcome (permanent disability/contribution to death)
**Recurrence of similar events**	
Remote	No known number of cases, 1 in 10,000
Low	Possible but no known number of cases, 1 case in 5000
Moderate	Documented but infrequent, 1 case in 200
High	Documented and frequent, 1 case in 100
Very high	Documented almost certain, 1 case in 20
**Error detectability**	
Very high	Error always detected, 9 out of 10 times the event happens
High	Error probably detected, 7 out of 10 times the event occurs
Medium	Moderate probability of detection, 5 out of 10 times the event occurs
Low	Low probability of detection, detecting 2 out of 10 times the event occurs
Remote	Almost impossible to detect, detecting 0 times out of 10 that the event occurs

## Data Availability

The data used and analyzed during the current study are available from the corresponding author upon reasonable request.

## References

[1] The New York Times Tracking Coronavirus Vaccinations around the World. https://www.nytimes.com/interactive/2021/world/covid-vaccinations-tracker.html.

[2] World Health Organization WHO Coronavirus (COVID-19) Dashboard. https://covid19.who.int/.

[3] European Centre for Disease Prevention and Control Overview of the Implementation of COVID-19 Vaccination Strategies and Deployment Plans in the EU/EEA. https://www.ecdc.europa.eu/en/publications-data/overview-implementation-covid-19-vaccination-strategies-and-deployment-plans.

[4] Wouters O.J., Shadlen K.C., Salcher-Konrad M., Pollard A.J., Larson H.J., Teerawattananon Y., Jit M. (2021). Challenges in ensuring global access to COVID-19 vaccines: Production, affordability, allocation, and deployment. Lancet.

[5] Liu T., He Z., Huang J., Yan N., Chen Q., Huang F., Zhang Y., Akinwunmi O.M., Akinwunmi B.O., Zhang C.J.P. (2021). A Comparison of Vaccine Hesitancy of COVID-19 Vaccination in China and the United States. Vaccines.

[6] Tatar M., Wilson F.A. (2021). The largest vaccination campaign in history: A golden opportunity for bundling public health interventions. J. Glob. Health.

[7] Italian Ministry of Health Patient Safety and Clinical Risk Management: Handbook for the Training of Health Professionals. https://www.salute.gov.it/imgs/C_17_pubblicazioni_640_allegato.pdf.

[8] Official Gazette of the Region of Veneto Resolution of the Regional Council No. 2255 of 30 December 2016. https://bur.regione.veneto.it/BurvServices/pubblica/DettaglioDgr.aspx?id=337226#:~:text=Semplificazione%20e%20riorganizzazione%20del%20modello,approvazione%20del%20programma%20delle%20attivit%C3%A0.&text=In%20materia%20di%20rischio%20clinico%20e%20sicurezza%20del%20paziente%20con%20la%20D.G.R..

[9] Centers for Disease Control and Prevention (CDC) and U.S Department of Health and Human Services. The Checklist of Best Practices for Vaccination Clinics Held at Satellite, Temporary, or Off-Site Locations. https://www.izsummitpartners.org/content/uploads/2019/02/off-site-vaccination-clinic-checklist.pdf.

[10] National Adult and Influenza Immunization Summit Pledge for Organizations Implementing Vaccination Clinics Held at Satellite, Temporary, or Off-site Locations 2018–2019 Influenza Season. https://www.izsummitpartners.org/content/uploads/2019/02/pledge-for-organizations-providing-vaccination-clinics.pdf.

[11] National Adult and Influenza Immunization Summit Ten Principles for Holding Safe Vaccination Clinics at Satellite, Temporary, or Off-Site Locations. https://www.izsummitpartners.org/content/uploads/2017/04/Ten-principles-for-safe-vac-clinics-1-pg-sum.pdf.

[12] Centers for Disease Control and Prevention Guidance for Planning Vaccination Clinics Held at Satellite, Temporary, or Off-Site Locations. https://www.cdc.gov/vaccines/hcp/admin/mass-clinic-activities/index.html.

[13] Official gazette of the Italian Republic Extension of the State of National Emergency and Further Measures to Contain the Spread of the COVID-19 Epidemic. https://www.gazzettaufficiale.it/eli/id/2021/12/24/21G00244/sg.

[14] Buja A., Manfredi M., De Luca G., Zampieri C., Zanovello S., Perkovic D., Scotton F., Minnicelli A., De Polo A., Cristofori V. (2021). Using Failure Mode, Effect and Criticality Analysis to Improve Safety in the COVID Mass Vaccination Campaign. Vaccines.

[15] Le Tohic S., Basso S., Peillard L. (2022). Cartographie des risques liés à l’organisation de la campagne de vaccination contre la COVID-19 [Risk mapping associated with organization of a COVID-19 immunization campaign]. Ann. Pharm. Fr..

[16] Institute for Safe Medication Practices Learning from Errors with the New COVID-19 Vaccines. https://www.ismp.org/resources/learning-errors-new-covid-19-vaccines.

[17] Institute for Safe Medication Practices Any New Process Poses a Risk for Errors: Learning from 4 Months of Coronavirus Disease 2019 (COVID-19) Vaccinations. https://www.ismp.org/resources/any-new-process-poses-risk-errors-learning-4-months-coronavirus-disease-2019-covid-19.

[18] Centers for Disease Control and Prevention COVID-19 Vaccine. Administration Errors Revaccination Guidance. https://www.cdc.gov/vaccines/covid-19/downloads/covid19-vaccine-errors-deviations.pdf.

[19] Italian Drug Agency FAQ—Pharmacovigilance on COVID-19 Vaccines. https://www.aifa.gov.it/domande-e-risposte-farmacovigilanza-vaccini-covid-19.

